# *Enterococcus faecium sagA* mutants have cell envelope defects influencing antibiotic resistance and bacteriophage susceptibility

**DOI:** 10.1128/jb.00302-25

**Published:** 2025-10-09

**Authors:** Garima Arya, Pavan Kumar Chodisetti, Juliel Espinosa, Brian C. Russo, Howard C. Hang, Breck A. Duerkop

**Affiliations:** 1 Department of Immunology and Microbiology, University of Colorado – Anschutz Medical Campus, School of Medicine, Aurora, Colorado, USA; 2 Department of Immunology and Microbiology, Scripps Research, La Jolla, California, USA; 3 Laboratory of Chemical Biology and Microbial Pathogenesis, The Rockefeller University 5929, New York, New York, USA; University of Illinois Chicago, Chicago, Illinois, USA

**Keywords:** antibiotic resistance, bacterial cell envelope, bacteriophage, *Enterococcus*, bacteriophage therapy, molecular genetics, peptidoglycan, cell wall, fluorescent image analysis

## Abstract

*Enterococcus faecium* is a gram-positive bacterium that is resident to the intestines of animals including humans. *E. faecium* is also an opportunistic pathogen that causes multidrug-resistant (MDR) infections. Bacteriophages (phages) have been proposed as therapeutics for the treatment of MDR infections; however, an obstacle for phage therapy is the emergence of phage resistance. Despite this, the development of phage resistance can impact bacterial fitness. Thus, understanding the molecular basis of fitness costs associated with phage resistance can likely be leveraged as an antimicrobial strategy. We discovered that phage-resistant *E. faecium* harbor mutations in the cell wall hydrolase gene *sagA*. SagA cleaves crosslinked peptidoglycan (PG) involved in PG remodeling. We show that mutations in *sagA* compromised *E. faecium* PG hydrolysis. One *sagA* mutant, with a defect in cell envelope integrity, increased cellular permeability, and aberrant distribution of penicillin-binding proteins, was also more sensitive to β-lactam antibiotics. These changes correspond to a growth defect where cells have abnormal division septa, membrane blebbing, and aberrant cell shape. The dysregulation of the cell envelope caused by the *sagA* mutation alters the binding of phages to the *E. faecium* cell surface, where phage infection of *E. faecium* requires phages to localize to sites of peptidoglycan remodeling. Our findings show that by altering the function of a single PG hydrolase, *E. faecium* loses intrinsic β-lactam resistance. This indicates that phage therapy could help revive certain antibiotics when used in combination.

**IMPORTANCE:**

*Enterococcus faecium* causes hospital-acquired infections and is frequently resistant to frontline antibiotics, including those that target the cell wall. Bacteriophages represent a promising alternative to combat such infections. However, bacterial adaptation to phage predation often results in resistance. Such resistance is frequently accompanied by fitness trade-offs, most notably altered antibiotic susceptibility. This study provides mechanistic insights into phage resistance-associated antibiotic sensitivity in *E. faecium*. We show that phage-resistant *E. faecium* carrying a mutation in the peptidoglycan hydrolase SagA has compromised cell envelope integrity, mislocalized penicillin-binding proteins, and become sensitized to β-lactam antibiotics. These findings highlight the potential of reviving antibiotics when used in combination with phages in the clinical setting.

## INTRODUCTION

Enterococci are intestinal gram-positive bacteria that cause severe systemic infection (1–4). Enterococcal bloodstream infections are associated with high mortality, and this has increased following the coronavirus disease 2019 pandemic (5, 6). Among medically relevant enterococci, *Enterococcus faecium* and *Enterococcus faecalis* are of particular concern because they are hospital-adapted and cause nosocomial infections. Nosocomial *E. faecium* infections are problematic due to the organism’s intrinsic and acquired resistance to diverse antibiotics, including β-lactams, aminoglycosides, and vancomycin (7–9). Notably, prior exposure to cephalosporins, a type of β-lactam, is a leading risk factor for *E. faecium* infection (10, 11). Additionally, a hallmark feature of *E. faecium* is evolved antibiotic resistance through the acquisition of foreign DNA via horizontal gene transfer, which has directly contributed to their ability to thrive as a hospital-adapted opportunistic pathogen (12, 13).

There is growing interest in developing approaches to limit enterococci in hospital settings, including targeted screening of at-risk patients, elevated hygiene practices, and limiting hospital stays (14–16). Aside from interventions in patient care, innovations in therapeutic approaches, including the use of bacteriophages (phages – viruses that infect bacteria) for the treatment of multidrug-resistant (MDR) enterococcal infections, are gaining traction (17–21). Despite the excitement surrounding the potential of phage therapy, phage predation can lead to phage-resistant bacteria during treatment (22–24). Although phage resistance has the potential to threaten the efficacy of phage therapies, there is mounting evidence that fitness constraints arise in phage-resistant bacteria, leading to trade-offs such as heightened antibiotic sensitivity, dampening of virulence, and colonization defects (25–29). Considering fitness trade-offs stemming from phage resistance are well-documented and could be exploited therapeutically, we know very little about molecular mechanisms that support these trade-offs.

Previously, our group discovered that *E. faecium* phage-resistant isolates are more susceptible to β-lactam antibiotics, including ampicillin and ceftriaxone (26). Sensitivity to cephalosporins is the result of mutations in SagA, a hydrolase that cleaves peptidoglycan (PG) during cell division (26, 30–33). Here, we leverage those strains to investigate the mechanistic basis for how mutations in SagA lead to β-lactam sensitivity in *E. faecium. E. faecium sagA* mutants exhibit diffuse penicillin-binding protein (PBP) localization throughout the periphery of the cell. Additionally, *sagA* mutants have cell-wall morphology defects characterized by mislocalized division septa, cell envelope protrusions, and aberrant cell shape that support phage resistance. Phages bind to discrete sites on the cell surface of *E. faecium* that are associated with active peptidoglycan synthesis, whereas phages are sequestered to cell envelope protrusions and cells with aberrant shape in the *sagA* mutant, resulting in non-productive phage infection. These findings provide a mechanistic basis for how SagA deficiency leads to β-lactam sensitivity and cell wall defects that support phage resistance. Intrinsic resistance to β-lactams is a common feature of *E. faecium,* and our findings support the idea of revisiting β-lactams as a potential treatment in combination with phage therapy.

## RESULTS

### The peptidoglycan hydrolase domain of *E. faecium* SagA is required for peptidoglycan cleavage and β-lactam resistance

**Fig 1:**
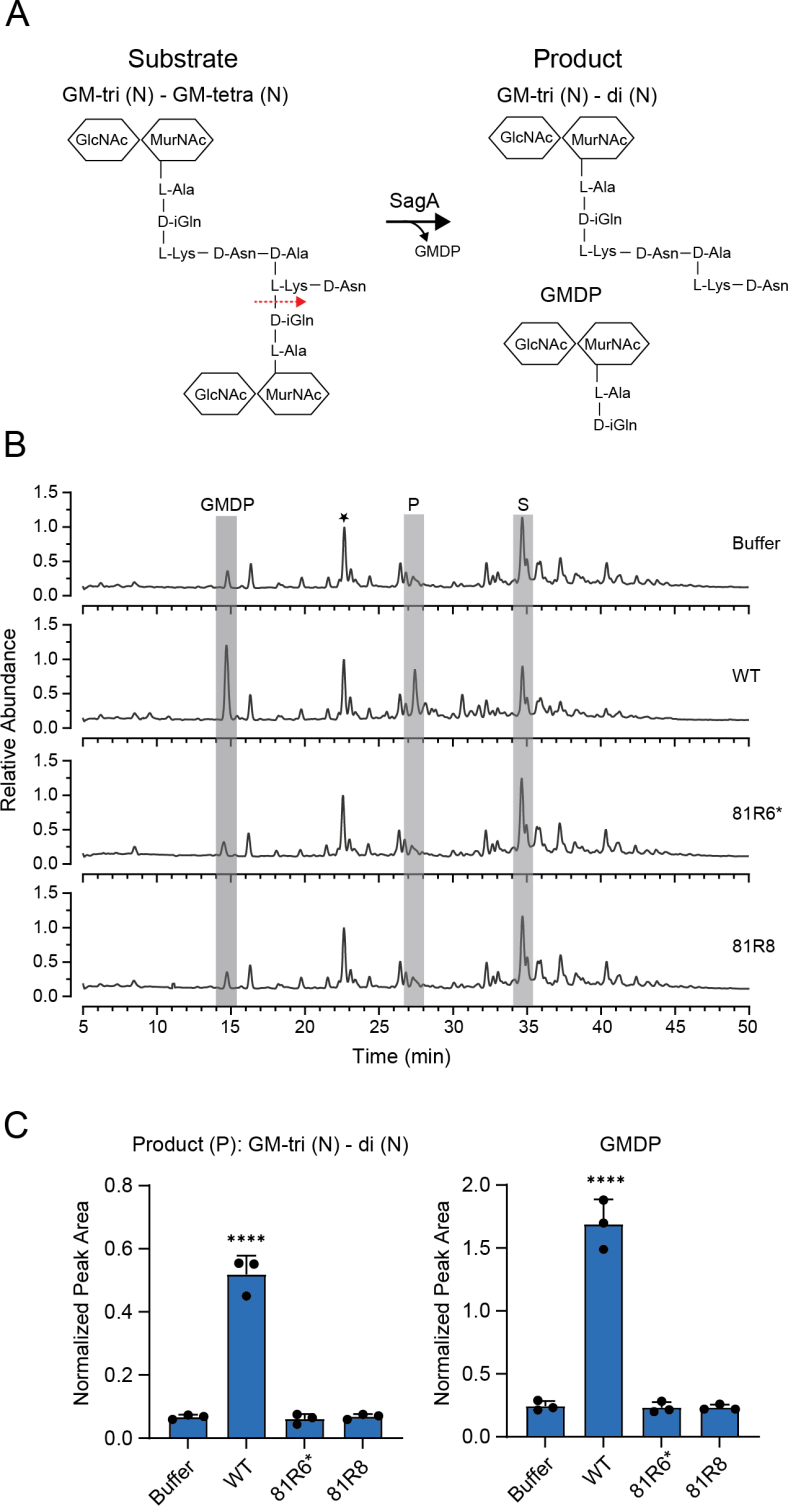
Phage-resistant sagA mutants have compromised D, L-endopeptidase activity. (**A**) Schematic showing SagA endopeptidase activity. Substrate denotes the crosslinked muropeptide released following mutanolysin digestion of intact *E. faecium* Com12 PG. SagA-mediated cleavage of substrate releases GMDP and Product: GM-tri (N) - di (N). (**B**) liquid chromatography-mass spectrometry (LC-MS) analysis of *E. faecium* Com12 PG incubated with purified SagA, SagAY451/L452-L ins (81R6*), SagA G435V (81R8). Total ion chromatogram was normalized to the intensity of unchanging GM-tri (D-Asn) peak marked with a star in the buffer control. P, product; S, substrate. (**C**) Quantification of Product and GMDP formation by SagA, SagAY451/L452-L ins (81R6*), SagA G435V (81R8). Extracted ion chromatograms of SagA-treated samples were compared to the buffer control by one-way analysis of variance with Dunnett’s post-test (*n* = 3). ****P < 0.0001. Comparisons with no asterisk had *P* > 0.05 and were not considered significant.

Prior work from our group revealed that *E. faecium* Com12 develops resistance to the phage 9181 by acquiring mutations in the *sagA* gene (26). *sagA* encodes an NlpC/P60 peptidoglycan hydrolase that cleaves crosslinked PG during cell wall remodeling (31, 33). Initially thought to be an essential gene (34), we recovered a variety of point mutations near critical amino acid residues in the NlpC/P60 hydrolytic domain, suggesting that these mutations rendered SagA non-functional. To test if these phage-mediated mutations in SagA hamper its PG hydrolytic activity, we assessed the *in vitro* enzymatic activity of *E. faecium* Com12 SagA from two independent phage-resistant mutant strains. Enzymatic activity was measured using mutanolysin-digested peptidoglycan from *E. faecium* Com12 and the production of N-acetylglucosamine (GlcNAc)-MurNAc-dipeptide (GMDP) (Fig. 1A). We initially chose to focus on two *E. faecium* mutant strains, 81R6 and 81R8, which have an F insertion between Y451 and L452 and a G435V substitution in the NlpC/P60 catalytic domain, respectively. Our attempts to purify the 81R6 SagA mutant version of SagA (F insertion between Y451 and L452) were unsuccessful. Therefore, to test if the position of this amino acid insertion is crucial for the catalytic activity of SagA*,* we made a version of SagA that has the smaller hydrophobic amino acid leucine inserted at this position. Both SagA mutants have a reduced ability to hydrolyze PG, suggesting that point mutations localized to the NlpC/P60 catalytic domain disrupt enzymatic activity (Fig. 1B and C). Consistent with this observation, the Hang lab recently showed that a chromosomal deletion of *sagA* could be made in the related *E. faecium* strain Com15, further confirming *sagA* to be a non-essential gene (33).

Our previous work also indicated that *E. faecium* SagA mutants were more susceptible to cell wall-targeting antibiotics and specifically cephalosporins, for which *E. faecium* is intrinsically resistant (35). Thus, to test the impact of SagA functionality on *E. faecium* resistance to cell wall-targeting antibiotics, we determined minimal inhibitory concentrations (MICs) using microbroth dilution assays against representative β-lactams including 2nd (cefuroxime)-, 3rd (ceftriaxone)-, 4th (cefepime)-, and 5th (ceftaroline)-generation cephalosporins, ampicillin, and meropenem, as well as the cyclic peptide bacitracin, which inhibits lipid carriers during PG synthesis (36). The *sagA* mutant strain 81R6 exhibited reduced MICs of at least twofold or greater for all antibiotics tested (Table 1), with sensitivity most enhanced for ceftriaxone (32-fold) and cefepime (128-fold). Importantly, ceftriaxone resistance was restored in *E. faecium* 81R6 by complementing *sagA* on a plasmid (Table S1). Our prior study confirmed that these SagA variants are expressed in *E. faecium* (26), indicating that an inability of SagA to effectively hydrolyze peptidoglycan results in sensitization to cell wall-targeting antibiotics. Together, these observations are consistent with our earlier findings, where loss of functional SagA results in antibiotic sensitivity (26, 33), and also extend this phenotype to a diversity of cell wall-targeting antibiotics.

**TABLE 1:** *sagA* mutant 81R6 has enhanced susceptibility to antimicrobials

	*E. faecium* Com12 MIC (µg/mL)^*a*^	
Antimicrobial	Wild type	81R6
Cefuroxime	512	128
Ceftriaxone	32	1
Cefepime	256	2
Ceftaroline	32	2
Ampicillin	4	1
Meropenem	64	32
Bacitracin	256	128

^*a*^ Median MICs determined from at least three independent replicates.

### Loss of SagA function compromises *E. faecium* cell wall integrity

**Fig 2:**
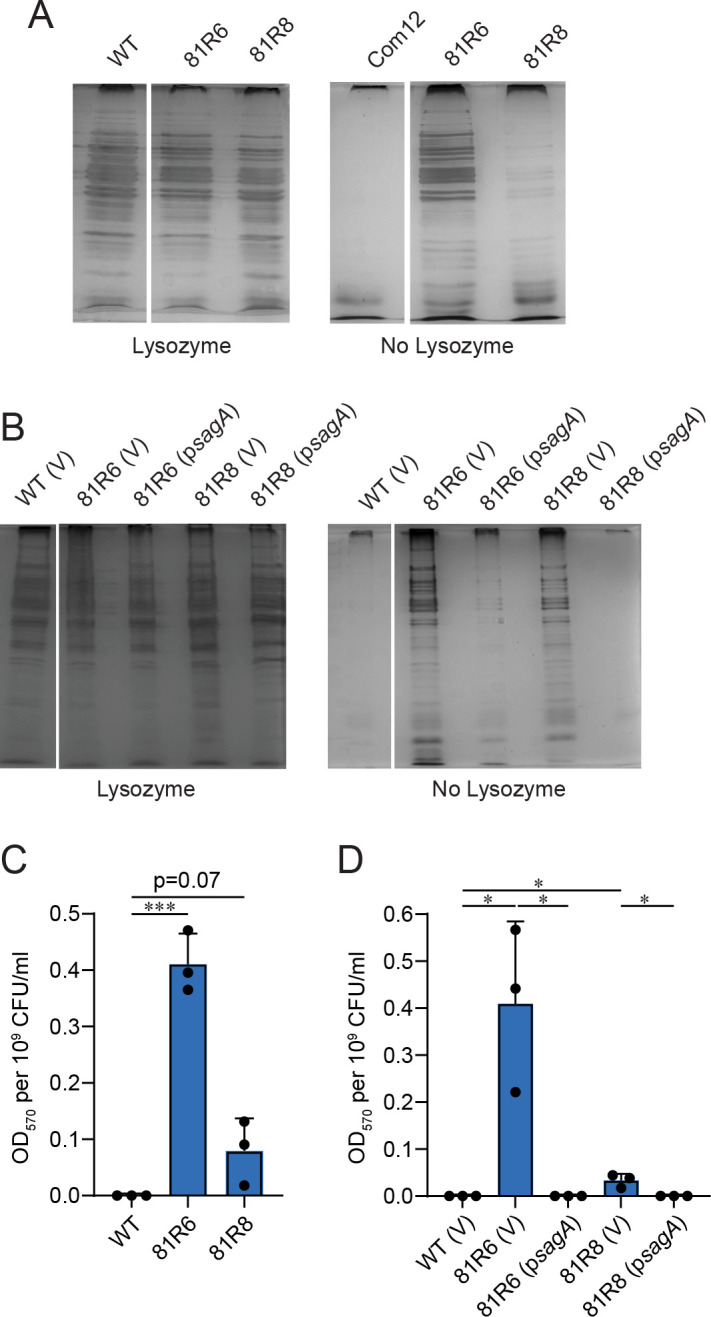
*E. faecium sagA* mutants exhibit compromised cell envelope integrity. (**A, B**) Cell envelope integrity of *E. faecium* Com12 (WT) and *sagA* mutant strains 81R6 and 81R8 strains. Strains carrying empty vector pAM401 (V) or pAM401 with Com12 *sagA* (p*sagA*). Exponentially grown cells in brain heart infusion broth were harvested and treated with (+) or without (−) lysozyme, prior to the addition of SDS Laemmli sample buffer. Samples were subjected to SDS-PAGE. Protein bands were visualized by silver staining. (**C, D**) Cell envelope damage for *E. faecium* strains described in A and B was assessed by measuring the hydrolysis of chlorophenol red-β-D-galactopyranoside (CPRG). CPRG hydrolysis was quantified after removing cells and measuring absorbance at 570 nm of the cell supernatant normalized to viable CFU. Data represent average (±SD) from three independent experiments. ND, not detected. *P*-values were calculated using unpaired two-tailed Student’s *t*-test (***P < 0.001; **P < 0.01; *P < 0.03).

Since SagA is involved in PG hydrolysis that supports symmetric cell division (33), we hypothesized that loss of *sagA* activity would result in compromised cell wall integrity. To test this, we exposed *E. faecium* Com12 and the *sagA* mutant strains 81R6 and 81R8 to 2% sodium dodecyl sulfate (SDS), an anionic detergent that disrupts membranes leading to bacterial cell lysis. When exposed to SDS, wild-type (WT) *E. faecium* experiences minimal cell lysis measured by the release of cytoplasmic protein (Fig. 2A). As a control, we first treated *E. faecium* Com12 with lysozyme to weaken the cell wall, and then exposed the cells to SDS, which was sufficient to lyse the cells (Fig. 2A). *E. faecium sagA* mutants, when treated with SDS alone, released high amounts of intracellular protein (Fig. 2A). Contrary to *E. faecium* Com12, both 81R6 and 81R8 *sagA* mutants released protein in the presence of only SDS. Notably, protein release by strain 81R6 in the presence of only SDS was comparable to that of wild-type cells treated with lysozyme and SDS. SDS resistance was restored by complementing *sagA* in the mutant strains (Fig. 2B). Together, these data indicate that loss of SagA function compromises *E. faecium* cell wall integrity.

Next, we sought to determine if the compromised cell wall integrity in these phage-resistant *sagA* mutants leads to an altered cell envelope. To test this, we performed an assay that requires intracellular β-galactosidase access to the impermeant small molecule substrate chlorophenol red-β-D-galactopyranoside (CPRG) (37). β-Galactosidase released from damaged cells hydrolyzes CPRG into the colored compound chlorophenol red, which can be quantified by measuring absorbance at 570 nm. *E. faecium* Com12 with an intact cell envelope is impermeable to CPRG, and no hydrolysis occurs (Fig. 2C). However, the *sagA* mutants 81R6 and 81R8 hydrolyzed CPRG to varying degrees, indicating a defective cell envelope and potentially increased permeability, leading to the leakage of endogenous β-galactosidase from the cells (Fig. 2C). Colony-forming units of both strains were similar across a series of optical densities (Fig. S1), suggesting that cell lysis is unlikely to account for β-galactosidase release. This cell envelope defect was restored when *E. faecium* 81R6 and 81R8 were complemented with *sagA* (Fig. 2D). These results show that *sagA* mutants have impaired cell wall integrity that may support increased permeability of the cell envelope.

### *E. faecium sagA* mutants exhibit increased penetration of β-lactams and mislocalized penicillin-binding proteins

**Fig 3:**
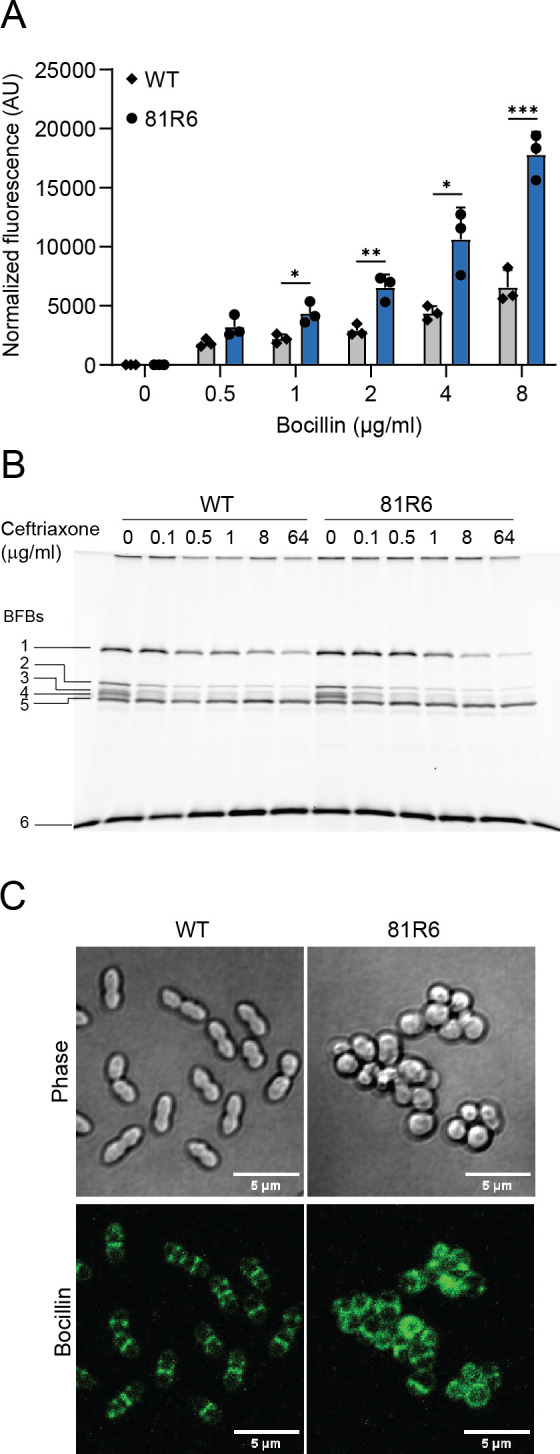
*In vivo* acylation of PBPs in *E. faecium sagA* mutants. (**A**) The *E. faecium sagA* mutant 81R6 is more reactive to Bocillin uptake. Fluorescence was measured at 488 nm and normalized to optical density (OD_600_) of the samples. Data represent average (±SD) from three independent experiments. ND, not detected. *P*-values were calculated using multiple comparisons *t*-test with adjusted *P*-values using Holm-Šidák method. ***P < 0.002; **P < 0.007; *P < 0.02. (**B**) Bocillin fluorescently labeled bands in the presence of ceftriaxone in the membrane fraction of *E. faecium* strains. Membrane fraction without ceftriaxone treatment was taken as control. BFBs (labeled as #1 to 6) were separated within SDS-PAGE gels, and bands were detected by imaging at 488 nm. The experiment was repeated at least three times. (**C**) Bocillin staining of *E. faecium* to show PBP distribution in the *sagA* mutant 81R6 compared to Com12 (WT) cells.

To determine if β-lactams could penetrate the *E. faecium* cell envelope more readily in the *sagA* mutants, we performed a β-lactam penetration assay using Bocillin, a fluorescent 4,4,-difluoro-4-bora-3a,4a-diaza-*s*-indacene (BODIPY)-labeled version of the β-lactam penicillin (38). Bocillin labels the PBPs of live cells and, like other β-lactams, including ceftriaxone, covalently binds to the active site of PBPs at the serine residue, thereby preventing transpeptidase activity. We focused our proceeding work on the *sagA* mutant strain 81R6, as this strain had a significant reduction in the ceftriaxone MIC (26) (Table 1) and experienced maximal damage to the cell envelope (Fig. 2C). Compared to *E. faecium* Com12, 81R6 showed increased uptake of Bocillin across different concentrations tested (Fig. 3A). Because ceftriaxone resistance in *E. faecium* is governed by the low affinity PBPs PbpA(2b) and Pbp5 and the class A PBPs PonA and PbpF (39, 40), increased cell envelope permeability in *E. faecium* 81R6 could lead to rapid PBP acylation by β-lactams, elevating ceftriaxone sensitivity. To assess this, we first treated actively growing cells with ceftriaxone to acylate available PBPs, followed by Bocillin to fluorescently label any PBPs that remained unoccupied by ceftriaxone. SDS-PAGE and fluorescent protein band imaging show that, in the absence of ceftriaxone, PBPs are readily acylated by Bocillin (Fig. 3B). In the presence of ceftriaxone, we did not observe any significant difference in the intensity of the Bocillin fluorescent bands (BFB1–6) between *E. faecium* Com12 and 81R6 (Fig. 3B). BFB1 shows high Bocillin signal in the absence of ceftriaxone, and this Bocillin signal decreases as the concentration of ceftriaxone increases. This indicates that this PBP has a higher affinity for ceftriaxone than Bocillin. For BFB2–4, a similar phenotype is observed. However, the Bocillin signal for BFB-5 and BFB-6 is not affected at the tested concentrations of ceftriaxone, indicating that these PBPs have low affinity for ceftriaxone, yet retain the ability to bind Bocillin. Together, these data indicate that the loss of cell envelope integrity of 81R6 results in no substantial impact on PBP acylation patterns.

For proper cell wall synthesis, PG hydrolases work in close association with PBPs (41, 42). SagA is a key PG hydrolase involved in PG remodeling, so it is possible that *sagA* mutations could alter the distribution of PBPs within the cell. To test this, we stained actively dividing *E. faecium* Com12 and 81R6 with Bocillin and visualized PBP localization using fluorescence microscopy. Wild-type cells show a diplococcus morphology with Bocillin labeling at the equator and division septa (Fig. 3C). In contrast, 81R6 cells showed mislocalized Bocillin staining throughout the periphery of the cell along with random sites of dense Bocillin accumulation (Fig. 3C). These data indicate that PBPs are disorganized in the absence of functional SagA and fail to localize properly during cell division.

### Peptidoglycan remodeling is altered in *E. faecium sagA* mutants

**Fig 4:**
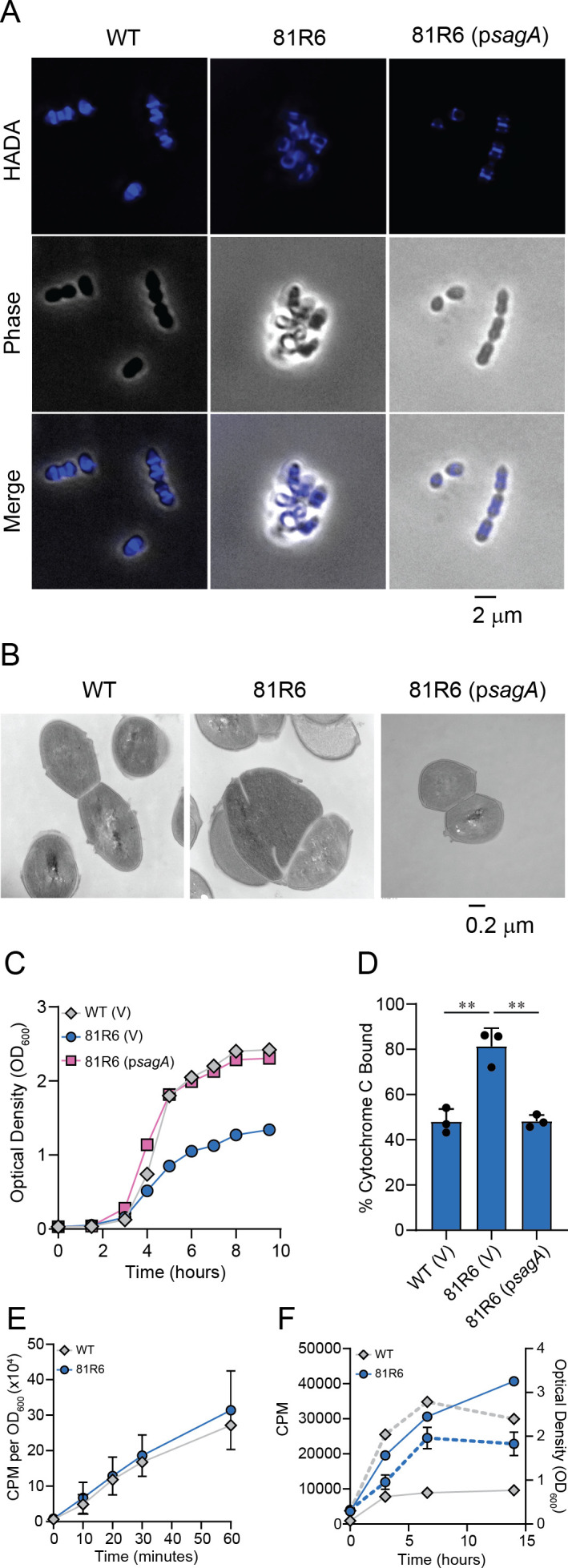
*E. faecium sagA* mutants show aberrant cell division and altered cell surface charge. (**A**) HADA staining of *E. faecium* Com12 (WT), 81R6, and 81R6 (*psagA*). (**B**) Transmission electron microscopy of *E. faecium* strains shown in A. (**C**) Growth curves of *E. faecium* Com12 (WT), 81R6, and 81R6 (*psagA*). V indicates strains carrying the empty vector pAM401. An experiment was performed three times, and a representative biological replicate is shown. (**D**) Cell surface charge of *E. faecium* Com12 (WT), 81R6, and 81R6 (*psagA*) using a cytochrome c binding assay. Unbound cytochrome c was quantified in the culture supernatant by measuring absorbance at 570 nm. Values represent mean ± SD of three biological replicates. *P*-values were calculated using unpaired two-tailed Student’s *t*-test. (***P < 0.001; **P < 0.01; *P < 0.03). (**E**) Incorporation of [^14^C]GlcNAc by *E. faecium* Com12 (WT) and 81R6. Values represent mean ± SD of three biological replicates. (**F**) Release of radiolabeled peptidoglycan fragments into the culture medium by *E. faecium* Com12 (WT) and 81R6. Values represent mean ± SD of three biological replicates. Solid lines indicate ^14^C signal measures at counts per minute using scintillation counting. Dashed lines indicate growth as measured by optical density (OD_600_).

SagA supports cell envelope integrity in *E. faecium*; thus, we hypothesized that mutating *sagA* would impair PG synthesis. To assess this, we analyzed *E. faecium* Com12 and 81R6 for their ability to incorporate 3-[[(7-hydroxy-2-oxo-2H-1-benzopyran-3-yl)carbonyl]amino]-D-alanine (HADA), a fluorescent analog of D-alanine. Using fluorescence microscopy, we observed that *E. faecium* Com12 cells showed HADA staining at the cell septa and equator, whereas 81R6 showed uniform distribution of HADA around the periphery of the cell, suggesting a defect in PG synthesis (Fig. 4A). Complementation of 81R6 with *sagA* restored the HADA staining pattern to that of wild-type cells (Fig. 4A). Transmission electron microscopy (TEM) confirmed that the 81R6 strain had an amorphous cell morphology with aberrant division septa, membrane blebbing, and aberrant cell shape (Fig. 4B). In line with these results, we observed a growth defect for 81R6 compared to *E. faecium* Com12, which could be restored upon complementation (Fig. 4C). A similar phenotype was observed for a *sagA* deletion strain of *E. faecium* Com15 (33), suggesting a role of functional SagA for proper cell division.

A connection between PG hydrolase activity and cell surface charge has been reported (43–45). Compromised cell envelope integrity and altered cell growth and division are hallmarks of cell envelope changes that can affect cell surface charge. Changes in cell surface charge can contribute to cell aggregation, a phenotype that is observed for 81R6 and has been shown for an *E. faecium sagA* clean deletion mutant (33). Thus, to determine if 81R6 has an altered cell surface charge, we tested the binding of the cationic protein cytochrome c to the bacterial cell surface. More cytochrome c bound to 81R6 than to wild-type *E. faecium* Com12, suggesting an increased negative cell surface charge (Fig. 4D). These data indicate that the *sagA* mutation corresponding to 81R6 alters the overall cell surface charge that likely contributes to the aggregation phenotype of 81R6.

We next assessed PG synthesis and turnover in *E. faecium* Com12 and 81R6. For PG synthesis, we monitored the incorporation of [^14^C]N-acetylglucosamine (GlcNAc) into exponentially growing cells. [^14^C]GlcNAc incorporation increases at the same rate for both *E. faecium* Com12 and 81R6 throughout growth (Fig. 4E), indicating that mutation of *sagA* does not affect the rate of PG synthesis under the conditions tested. To evaluate PG turnover, we measured the release of radiolabeled PG fragments into culture supernatants. Cells were grown in media containing [^14^C]GlcNAc, washed, and resuspended in media containing unlabeled GlcNAc. The amount of free radioactivity in the culture media was measured over time. PG release occurred during the first 2.5 h of growth for *E. faecium* Com12 and then halted leading up to stationary phase (Fig. 4F). In contrast, 81R6 released PG much more rapidly and for a longer period of time, indicating that possible cell lysis in the stationary phase occurs as a consequence of compromised cell envelope integrity (Fig. 4F).

### SagA directs phages to adsorb to the cell surface at sites associated with cell division

**Fig 5:**
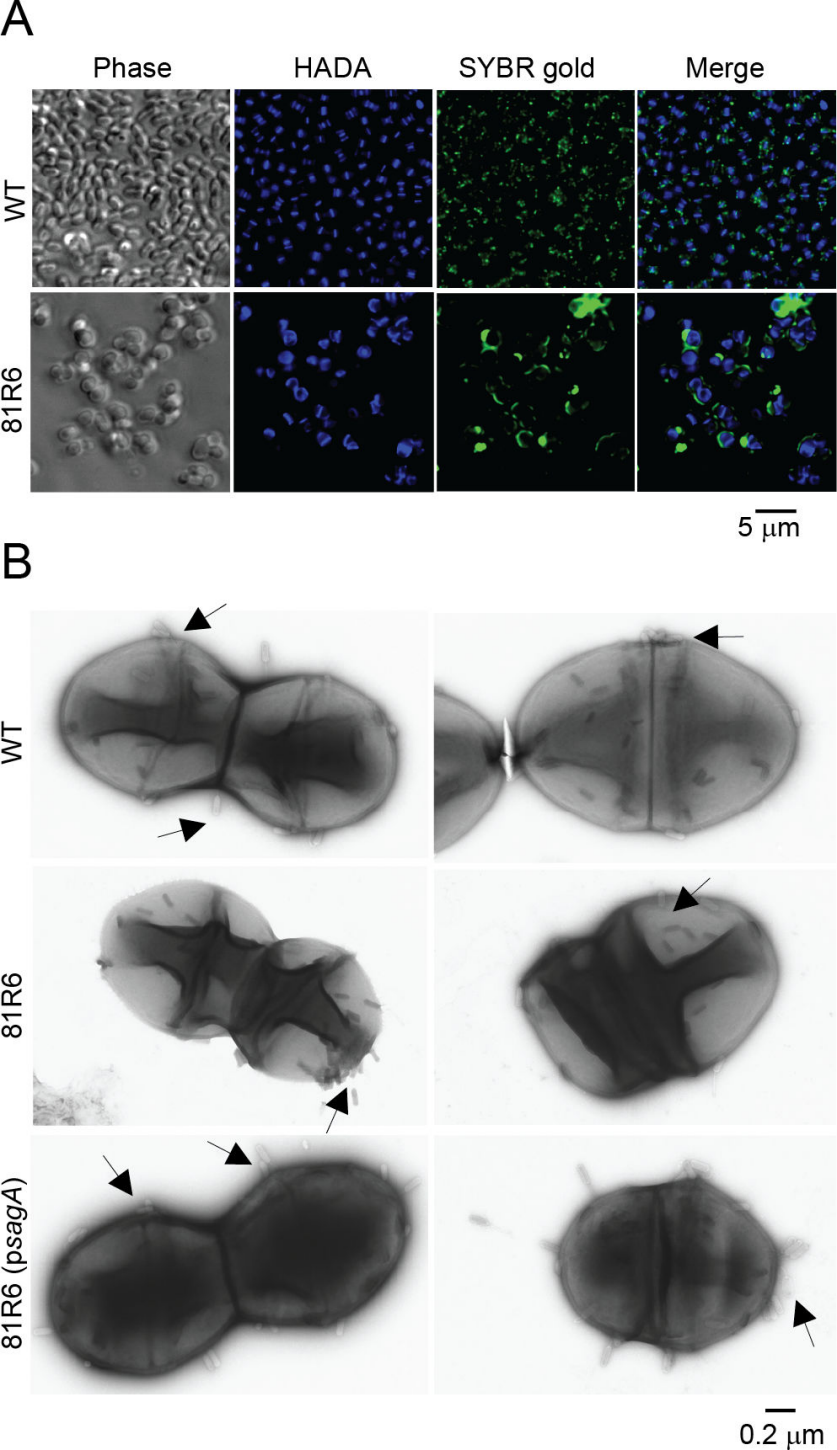
Binding of phage 9181 particles to the surface of *E. faecium*. (**A**) Fluorescence microscopy of *E. faecium* Com12 (WT) and 81R6 shows differential phage adsorption. HADA-stained Com12 (WT) and 81R6 cells were mixed with SYBR Gold-labeled phage 9181 particles. Cells were visualized using fluorescence microscopy 5 min post-infection. The experiment was repeated two times. Representative images are shown. (**B**) Visualization of phage adsorption on *E. faecium* strains by transmission electron microscopy. *E. faecium* Com12 (WT), 81R6, and 81R6 (p*sagA*) strains were mixed with phage 9181 particles and incubated for 5 min at room temperature to allow the phages to attach to the bacterial cells. Phage particles are shown with arrows. At least 15 individual images were captured, and the experiment was repeated two times. Representative images from single experiments are shown.

To adsorb to bacterial cells, phages bind to surface molecules such as polysaccharides, carbohydrate components, and integral membrane proteins that are positioned in spatially distinct locations of the cell envelope (46, 47). Previously, we showed that phages require SagA for infection and that phage adsorption to the *E. faecium* cell surface was unaffected by *sagA* mutation (26). Therefore, it is likely that compromised cell wall integrity and improper cell division in *sagA* mutants alters the spatial distribution of phage receptors that supports non-specific phage adsorption and prevents DNA entry into cells. Thus, we fluorescently labeled phage 9181 with SYBR Gold and assessed its distribution at the surface of *E. faecium* and 81R6 cells using fluorescence microscopy. *E. faecium* showed punctate SYBR Gold signal across the cell surface with slightly enhanced intensity at locations that correlated with sites of HADA incorporation (Fig. 5A). Approximately 35% of the phage puncta localized to HADA-positive regions, which indicate the division septa and cell equator. Notably, these HADA-positive regions account for 18% of the total cell area. Phage association with septa and equator regions is nearly twofold higher than would be expected based on area alone. Interestingly, the majority of 81R6 cells showed faint SYBR Gold signal at the cell surface and very intense localized signal at altered sites of cell division, near cells with aberrant shape, and membrane blebs (Fig. 5A). We posit that this intense concentrated signal could be due to mislocalized and sequestered phage receptors or phage DNA ejected into cell compartments that fail to separate during cell division (Fig. 4B). These differences in phage 9181 phage adsorption were confirmed by transmission electron microscopy showing that *E. faecium* Com12 supported phage adsorption at or near the cell equator and septa (Fig. 5B). In contrast, 81R6 cells showed phage aggregation at discrete locations along with phage distribution across the cell surface (Fig. 5B). Localization of phages at the cell surface was restored to a wild-type pattern by *sagA* complementation (Fig. 5B). Collectively, these results suggest that phage receptors coordinate phage binding and infection across the cell surface with some preference for active sites of cell division.

## DISCUSSION

Multidrug-resistant enterococci and the treatment challenges associated with their infections have renewed attention toward the development of phage therapies. The emergence of phage resistance represents a potential hurdle for successful phage therapies. However, bacteriophage resistance can result in fitness costs, including antibiotic sensitization, colonization defects, and reduced virulence (20, 25, 48–53). Importantly, these fitness costs could be exploited for therapeutic advantage in the clinic; thus, extensively characterizing the mechanistic basis of such fitness trade-offs warrants further study. In the present work, we explore β-lactam sensitivity in *E. faecium* lacking functional SagA, a peptidoglycan hydrolase required for phage infection (26). SagA mutations that support phage resistance lie in the NlpC/P60 hydrolase domain and impair the PG hydrolytic activity of SagA, similar to previously shown SagA catalytic domain mutants that are non-functional (31). One particular sagA mutation in strain 81R6 results in compromised cell wall integrity, increased cellular permeability, diffuse distribution of PBPs, and sensitivity to β-lactam antibiotics including cephalosporins. Additionally, *E. faecium sagA* mutants showed irregular distribution of newly synthesized PG in their cell wall and amorphous cell shape. These cell wall synthesis deficiencies further support the observation of cephalosporin sensitivity and show that by altering a critical PG hydrolase, *E. faecium* loses intrinsic cephalosporin resistance. Non-functional SagA also appears to affect the spatial distribution of phage receptors, supporting a role for SagA in mediating phage infection. To date, detailed studies exploring SagA and its connection with phage and antibiotic resistance in *E. faecium* are limited. Our findings provide insights into the connection between an antibiotic resistance fitness cost resulting from phage selective pressure that could possibly inform approaches to revive cephalosporins as anti-*E*. *faecium* drugs.

We discovered that *sagA* mutants had cell wall integrity defects and heightened sensitivity to β-lactam antibiotics, specifically cephalosporins. Our data show that the *sagA* mutant strain 81R6 has a compromised cell envelope with increased permeability to small molecules including β-lactams. β-Lactams inactivate PBPs and thereby kill bacteria by inhibiting cell wall synthesis (54). The efficacy of a given β-lactam in inhibiting PG crosslinking usually depends on its reactivity toward the entire repertoire of PBPs expressed by bacterial cells. Despite having deficiencies in cell envelope integrity, PBPs in the *sagA* mutant 81R6 were occupied by β-lactams similarly to those from wild-type *E. faecium* in the presence of a competing β-lactam (ceftriaxone). We did observe that the *sagA* mutant 81R6 has altered PBP distribution that may affect proper PBP localization and function, leading to cell wall synthesis deficiencies. Notably, the high-molecular-weight PBPs PbpA and Pbp5 are required for intrinsic ceftriaxone resistance in *E. faecium* and *E. faecalis* (39, 40). A variety of phenotypes similar to those of the *sagA* mutant strain 81R6 are shared with these PBP mutants, including impaired cell wall integrity, reduced growth rate, and aberrant cellular morphology (40). Thus, the role of SagA in supporting intrinsic β-lactam resistance likely lies in its ability to control the distribution of PBPs to sites of active cell division. When disrupted, PBPs such as low-affinity PBPs that are intrinsically resistant to β-lactams may fail to localize to the correct sites of cell division, resulting in a growth defect that supports enhanced β-lactam activity during cell wall synthesis.

Hydrolases like SagA fulfill cellular PG remodeling demands and maintain the structural integrity of cells during growth. Their disruption can lead to uncontrolled PG degradation and autolysis (43). HADA incorporation at the cell division septa and equator into actively dividing *E. faecium* cells suggested a role for SagA in PG remodeling (31). In line with this, 81R6 cells displayed aberrant HADA staining and distorted shape, indicating that functional SagA is crucial for proper cell shape and PG remodeling. Specifically, 81R6 showed misplaced septa and bulged cellular morphology, indicating improper cell division. PG hydrolases are also required for controlled cell wall synthesis and release PG fragments during cell growth and during PG turnover (43). The *sagA* mutant strain 81R6 showed increased release of radioactive GlcNAC, indicating altered PG turnover. Despite the alteration of PG turnover, 81R6 does not have a deficiency in PG synthesis. The increased PG turnover by 81R6 may be attributed to cell lysis due to compromised cell wall integrity and/or increased levels of muropeptides as observed in a similar *sagA* deletion strain (33). Thus, functional *sagA* is required for the controlled release of PG fragments during normal PG turnover.

Bacterial cell growth and division are intimately linked to PG remodeling. PBPs coordinate with the divisome, a complex of proteins involved in PG synthesis, membrane constriction, and cell division (55–58). In several bacterial species, SEDS (shape, elongation, division, sporulation) family proteins work with specific PBPs to mediate PG synthesis at specific positions in the cell (59–61). A recent PG synthesis model for enterococci showed that specific PBPs or PG synthases are localized at the site of cell division and elongation that are involved in septal PG and peripheral PG synthesis during growth, respectively (40, 62). Enterococcal PbpA(2b) and Pbp5, which are required for growth in the presence of cephalosporins, are proposed to catalyze PG synthesis at distinct cellular sites (40). Consistent with these findings, our Bocillin labeling experiments showed that PBPs are localized at the equator and septum in wild-type cells, suggesting their participation in septal and peripheral PG synthesis (Fig. 3C). In contrast, dispersed labeling of Bocillin in the *sagA* mutant 81R6 suggests PBPs are mislocalized, leading to growth and division defects. PBPs and cell division proteins work in a coordinated manner for proper PG synthesis. Key cell division proteins interact with different components of the PG synthesis machinery. For instance, FtsZ coordinates with PBPs during Z-ring formation (58), and the FtsEX complex coordinates with PG hydrolases like SagA for proper cell division (63–65). The aberrant septa and unseparated daughter cells observed for 81R6 suggest that the loss of SagA function likely leads to divisome assembly problems. Based on our findings, we can infer that SagA works in close association with cell wall synthases, cell division, and elongation machinery, and disruption of these interactions likely contributes to the β-lactams sensitivity and phage resistance of *sagA* mutants. However, additional studies are required to further prove a role for SagA in maintaining the fidelity of the divisome and elongasome machinery.

Our study highlights the cost of phage resistance in *E. faecium* resulting in pleiotropic changes in phenotypic traits that promote antibiotic sensitivity. However, this largely depends on the type of phage and how the host responds to the phage-imposed selective pressure. In gram-positive bacteria, the ejection of phage DNA requires penetration of the PG barrier and interaction with the membrane. This is a two-step process where phages first bind to carbohydrate-rich molecules to position themselves in proximity to a phage receptor at the cell surface, followed by interaction with the cell membrane (66–68). In enterococci, evidence of this process exists for *E. faecalis* where a transmembrane phage infection protein (PIP_EF_) is required for phage DNA entry but not for the initial phage adsorption that is driven by a surface-exposed exopolysaccharide (48, 49). We show here that the phage resistance phenotype in *sagA* mutants is not caused by an adsorption defect but most likely by altered phage receptor localization or modifications to the cell membrane that do not support phage DNA ejection. As *sagA* mutants have cell membrane abnormalities, a membrane-embedded receptor could be mislocalized or sequestered to membrane blebs or cells with aberrant shape that do not support phage DNA entry or replication. However, it is also possible that phage selective pressure imposed on bacteria could first destabilize the membrane, which indirectly leads to the mutations in *sagA*.

In conclusion, this study provides insights into how phage-mediated pressure, which results in phage resistance mutations, alters the cell envelope of *E. faecium,* leading to antibiotic sensitization. Our findings reveal that the cell wall hydrolase SagA, which supports phage infection (26), also plays a crucial role in maintaining cell wall integrity. Our data also provide insight into the role of SagA in coordinating PBP function that influences cephalosporin resistance. Therefore, phages could be used to select for *E. faecium sagA* mutants that could be more efficiently targeted with cephalosporin therapies. Such a phage-based strategy would be beneficial to help overcome the intrinsic cephalosporin resistance that plagues antibiotic therapies employed against this important nosocomial pathogen.

## MATERIALS AND METHODS

### Bacterial strains, bacteriophages, and growth conditions

Bacterial strains and bacteriophages used in this study are listed in Table S2. *E. faecium* strains were cultivated in brain heart infusion (BHI) broth at 37°C with shaking at 220 rpm. *Escherichia coli* strains were propagated in lysogeny broth (LB) at 37°C with shaking at 220 rpm. Chloramphenicol was added to media at 10 µg/mL or 5 µg/mL for maintenance of plasmids in *E. coli* and *E. faecium,* respectively.

### SagA mutant construction and protein purification

Site-directed mutagenesis of the *E. faecium* 81R6 and 81R8 SagA mutants in the pET-21a(+) vector (Millipore Sigma) was performed using Q5 Hot Start High-Fidelity 2X Master Mix (New England Biolabs, NEB), following the manufacturer’s instructions. To generate signal sequence deletion mutants of full-length 81R6 and 81R8 SagA, *E. faecium* Com15 SagA-His_6_-pET-21a(+) (69) was used as a template along with the primer pairs listed in Table S2. Constructs were transformed into *E. coli* Dh5α (NEB) according to the manufacturer’s protocol (ampicillin, 100 µg/mL). Clones were verified by Sanger sequencing, and confirmed pET-21a(+) plasmids were transformed into *E. coli* BL21-CodonPlus (DE3)-RIL (Agilent) according to the manufacturer’s instructions for isopropyl-β-D-thiogalactopyranoside (IPTG)-inducible expression (ampicillin, 100 µg/mL and chloramphenicol, 25 µg/mL). Signal sequence deletion mutants of full-length 81R6, 81R8, and *E. faecium* Com15 SagA were expressed and purified as previously described (30, 31).

### Purification and mutanolysin digestion of PG

Enterococci were grown in BHI medium with shaking at 37°C to log phase (OD_600_ of 0.6). PG was extracted by resuspending the bacterial cell pellet in 0.25% SDS solution in 0.1 M Tris-HCl, pH 6.8, and boiling the suspension for 20 min at 100°C. The resulting insoluble cell wall preparation was washed with distilled water six times until free of SDS. The cell wall was purified by treatment with benzonase followed by trypsin digestion. Then, the insoluble cell wall was recovered by centrifugation (16,000 × *g*, 10 min, 4°C) and washed once in distilled water. To obtain pure PG, cell wall was suspended in 1 M HCl and incubated for 4 h at 37°C to remove wall teichoic acids. The insoluble material was collected by centrifugation (16,000 × *g*, 10 min, 4°C) and washed with distilled water repeatedly until the pH was 5–6. The final PG was lyophilized and stored at −20°C. For muropeptide analysis, 2 mg purified PG was digested with 1 mg/mL mutanolysin from *Streptomyces globisporus* (Sigma, 10 KU/mL of mutanolysin in ddH_2_O) in 10 mM sodium phosphate buffer, pH 4.9, for 16 h at 37°C. The enzyme reaction was stopped by incubating at 95°C for 5 min.

### SagA PG hydrolase activity

To characterize the PG hydrolase activity of SagA and its variants, 100 μg–200 μg of mutanolysin-digested PG was incubated with 10 µM of purified protein in 50 mM Bis-Tris, pH 5.5, for 16 h at 37°C. Reactions were quenched by boiling at 95°C for 5 min and centrifuged at 16,000 × *g* for 5 min at room temperature (RT) to remove precipitated protein. Supernatants containing soluble muropeptides were isolated and lyophilized before processing for LC-MS analysis.

### LC-MS analysis of PG fragments

Lyophilized muropeptides were resuspended in 20 µL of 250 mM NaH_2_BO_3_, pH 9.0, and treated with ~1 mg NaBH_4_. Tubes were shaken until completely dissolved and then incubated for 1 h at room temperature. Reactions were quenched by adding 10 µL of 20% H_3_PO_4_ and incubating for 1 h at room temperature. Reduced samples were centrifuged at 16,000 × *g* for 10 min, and supernatants were transferred to LC-MS vials. For each sample, 10 µL was injected onto a C18 reverse-phase column (Acclaim 120 C18, 3 µm, 2.1 × 150 mm [DX059130]) at 176 µL/min and kept at 52°C using an isocratic gradient. The molecules were eluted using a gradient increasing from 0% B/100% A to 100% B/0% A in 59 min (Buffer A: 0.1% trifluoroacetic acid [TFA], Buffer B: 30% methanol/0.1% TFA). Solvent composition was held at 100% B/0% A for 5 min, after which the column was conditioned for 5 min at 0% B/100% A. All solvents were LC-MS grade.

Reverse phase-separated molecules were measured in MS mode using an Orbitrap XL operated at 60,000 resolution (auto gain control [AGC] of 1–2 × 10^5^ and maximum injection time of 500 ms). Collision-induced dissociation MS/MS spectra were acquired in ion trap mode (AGC of 1 × 10^4^ and maximum injection time of 25 ms) fragmenting one or two of the most abundant ions per cycle. Total ion and extracted ion chromatograms were analyzed using Xcalibur and Skyline. Molecular masses of PG fragments were determined using ChemDraw.

### Antimicrobial susceptibility assay

MICs of antibiotics were determined using microbroth dilution assays. Overnight-grown *E. faecium* strains normalized at an OD_600_ of ~5 × 10^5^ CFUs were inoculated into 96-well plates containing twofold serial dilutions of antibiotics in BHI medium (with chloramphenicol for plasmid maintenance when required). Plates were incubated at 37°C with shaking at 220 rpm for 24 h. OD_600_ was measured at different intervals, and the lowest antibiotic concentration that showed no growth was noted as the MIC.

### Cell wall integrity analysis

Cell wall integrity was analyzed by testing the susceptibility of intact *E. faecium* cells to SDS. Briefly, for testing SDS-mediated lysis, overnight grown cells were inoculated into BHI medium and grown to exponential phase (OD_600_ ~0.2). Cells were harvested and resuspended in 50 µL lysozyme buffer (10 mM Tris, pH 8.0, 50 mM NaCl, 20% sucrose). Cell suspensions were split into two equal aliquots. One aliquot was treated with 5 mg/mL lysozyme. All the samples were incubated at 37°C for 5 min. SDS Laemmli buffer (1M Tris-HCl [pH 6.8), 2% SDS, 50% glycerol, 25% β-mercaptoethanol, 0.02% bromophenol blue) was added, samples were heated at 95°C for 5 min and run on 12% mini SDS-PAGE gels at 110 V. PAGE gels were stained with Pierce Silver Stain (Thermo Fisher) according to the manufacturer’s protocol and visualized using a SYNGENE G:BOX Chemi XX6 gel doc imaging system.

To assess cell envelope permeability via the β-D galactopyranoside (CPRG) hydrolysis assay, *E. faecium* cultures were grown to stationary phase (~9 h) in BHI medium supplemented with 40 µg/mL of CPRG (Millipore Sigma) at 37°C. Cells were removed by centrifugation, and the supernatant was assessed for (CPRG) hydrolysis by measuring absorbance at 570 nm.

### PBP acylation assays

Overnight grown cells were diluted to an OD_600_ of 0.03 in BHI medium and grown for 4.5 h at 37°C with shaking at 220 rpm. One milliliter aliquots of cultured cells were harvested by centrifugation, washed, and resuspended in phosphate-buffered saline (PBS). Cell suspensions were incubated with varying concentrations (0, 0.5, 1, 2, and 4 µg/mL) of Bocillin (Thermo Fisher) at 37°C for 30 min. Cells were collected by centrifugation, washed, and resuspended in PBS. Cells were diluted fourfold, and 200 µL was dispensed in triplicate in 96-well plates. Fluorescence was measured using a microplate reader in top mode with excitation and emission at 488 and 517 nm, respectively. Fluorescence was normalized to OD_600_.

For PBP acylation assays, cultures were grown as described above. Cells were treated with ceftriaxone (Millipore Sigma) (0, 0.1, 0.5, 1, 8, 64 µg/mL) for 30 min at 37°C with shaking at 220 rpm. Cells were collected by centrifugation at 9,400 × *g* for 10 min, resuspended in PBS, normalized to an OD_600_ of 8.0 in 5 mL, and treated with Bocillin (1 µg/mL) for 30 min at 37°C. Cells were washed twice with PBS, resuspended in 3 mL of PBS, and disrupted by bead beating using Lysing Matrix B tubes (MP Biomedicals) for 10 min by placing samples on ice for 2 min after every cycle. Supernatant was collected by centrifugation at 21,130 × *g* for 10 min at 4°C. The membrane fraction was collected by ultracentrifugation at 100,000 × *g* for 30 min. Protein concentration was analyzed by Bradford assay using Bio-Rad protein assay dye reagent concentrate. Samples with equivalent protein concentrations were subjected to 10% SDS-PAGE using 20 cm gels at 140 V for 40 h at 4°C. Gels were scanned for Bocillin-labeled protein bands at 488 nm using a Bio-Rad ChemiDoc MP imaging system.

### Microscopy

To visualize *in vivo* localization of PBPs, *E. faecium* cells were stained with Bocillin. Exponential-phase (~0.4–0.5 OD_600_) cells were incubated with 5 µM Bocillin in BHI media for 30 min at 37°C. Cells were washed twice with PBS and fixed with 1% formaldehyde for 15 min at RT. Cells were washed and resuspended in PBS. For imaging, cells were mounted on 1% agarose pads and imaged under a Zeiss LSM 780 confocal laser scanning microscope.

To assess *in vivo* PG synthesis using HADA (Tocris Bioscience), exponentially grown (0.4–0.5 OD_600_) cells were collected by centrifugation at 9,400 × *g* and resuspended in BHI. Cells were incubated with 2.5 mM HADA at 37°C for 40 min, followed by washing with PBS. Cells were fixed with 3.7% paraformaldehyde. Cells were seeded onto 22 × 22 mm coverslips. For mounting, 10 µL of ProLong Diamond Antifade Mounting Media (Thermo Fisher) was placed on slides, and coverslips were laid on slides. Slides were incubated in the dark overnight and imaged using a Nikon Eclipse Ti-2 inverted light microscope equipped with an Orca Fusion BT cMOS camera (Hammamatsu), an IRIS 15 cMOS camera (Photometrics), and Semrock BrightLine filters.

To visualize phage 9181 adsorption to HADA-labeled *E. faecium* cells, phage 9181 was purified as described (26). A total of 100 µL of purified phage particles of 10^10^ plaque forming units (PFU)/mL were mixed with SYBR Gold nucleic acid stain (Thermo Fisher) at a 10,000:5 (vol/vol) ratio and incubated at RT for 15 min. The mixture was washed with SM-plus buffer (100 mM NaCl, 50 mM Tris-HCl, 8 mM MgSO_4_, 5 mM CaCl_2_ [pH 7.4]) three times using a 3 kDa cutoff Amicon filter (Millipore Sigma). Exponentially grown *E. faecium* cells (OD_600_ 0.4) were collected, washed, and resuspended in BHI and incubated at 37°C with 2.5 mM HADA for 40 min. After two washes, cells were resuspended in PBS and mixed with SYBR Gold-labeled phages (1:1), incubated for 5 min at RT, washed in SM-plus buffer, and transferred to poly-L lysine-coated microscope slides. Cells were imaged using a Nikon Eclipse Ti-2 inverted light microscope as described above. To quantify phage particles associated with HADA-positive staining, a machine learning algorithm was trained using Segment.ai in NIS Elements (Nikon) following manual drawing of regions of HADA-positive signal on a training data set. Whole bacteria were identified by FM4-64 staining (Thermo Fisher, channel not shown in Fig. 5) using standard segmentation analysis, and phages were identified using a bright spot detector with the General Analysis 3 module in NIS Elements. Total phage particles present in the images were counted, as were the number of phage particles that associated with bacterial cells or with HADA-positive regions of the bacteria.

For transmission electron microscopy, samples were prepared as described previously (70). Briefly, cells were grown in BHI medium to an OD_600_ of 0.4, and 1.5 mL of cells was collected by centrifugation. Cells were washed with cacodylate buffer (CB) (0.1 M, pH 7.4) and fixed at 4°C with 150 µL of fixative buffer containing formaldehyde (2%) and glutaraldehyde (2%) in CB. Samples were submitted to the University of Colorado Anschutz Medical Campus Electron Microscopy Core Facility for imaging. For negative staining, exponentially grown cells were collected by centrifugation, resuspended in SM-plus buffer, and incubated with purified phage 9181 for 5 min at RT. Cells were washed with SM-plus, resuspended in fixative buffer, and submitted for electron microscopy imaging. Ten microliters of sample was applied to glow-discharged 300-mesh formvar- and carbon-coated grids (Electron Microscopy Sciences) for 5 min and blotted with filter paper. The grid was washed one time by applying 10 µL of water to the grid and blotting with filter paper. The grids were stained with 10 µL of 0.5% uranyl acetate. Samples were imaged on a Thermo Fisher Tecnai G2 Biotwin TEM at 120 kV with an AMT low-mount NS15B sCMOS camera (AMT Imaging).

### Growth curves

Stationary phase cells were inoculated into BHI medium to an OD_600_ of 0.03, and cultures were incubated at 37°C with shaking at 220 rpm. OD_600_ was measured using a Genesys 30 visible spectrophotometer (Thermo Fisher).

### [^14^C]GlcNAc incorporation

Peptidoglycan synthesis was assessed by the incorporation of [^14^C]GlcNAc (PerkinElmer) in exponentially growing cells as described previously with minor modifications (40). Bacteria were grown in BHI medium to an OD_600_ of 0.2 at 37°C with shaking. Cultures were diluted to an OD_600_ of 0.03 in prewarmed BHI medium supplemented with 0.2 µCi/mL [^14^C]GlcNAc. Four hundred microliter aliquots were collected, washed with 1 mL PBS, and cell pellets were suspended in 150 µL water. Cells were transferred to 4 mL of Econo-Safe scintillation fluid (Thermo Fisher), and radioactivity was measured by scintillation counting using a Beckman LS6500 scintillation counter. Optical density was measured from parallelly grown label-free cells, and data are represented as counts per minute per OD_600_.

### Measurement of ^14^C release

Cells were labeled with [^14^C]GlcNAc as described above, collected by centrifugation, washed with PBS, and resuspended in BHI medium containing label-free 1 mM GlcNAc (Millipore Sigma). Aliquots were withdrawn, cells were pelleted, and the amount of released radioactivity in supernatant was measured by scintillation counting.

### Cytochrome c binding assay

Bacterial cell surface charge was analyzed as described previously (70). Briefly, BHI-grown stationary-phase cells were collected, washed twice with 20 mM morpholinepropanesulfonic acid buffer, pH 7.0, and normalized to an OD_600_ of 7.0. Nine hundred microliters of cell suspension was mixed with 100 µL of 5 mg/mL cytochrome c (Millipore Sigma) and incubated for 30 min. Cells were pelleted, and the absorbance of the supernatant was measured at 530 nm.

## SUPPLEMENTAL MATERIAL

Supplemental tables and figure jb.00302-25-s0001.pdf Tables S1 and S2, and Figure S1.

ASM does not own the copyrights to Supplemental Material that may be linked to, or accessed through, an article. The authors have granted ASM a non-exclusive, world-wide license to publish the Supplemental Material files. Please contact the corresponding author directly for reuse.
