# Supplementary material for: Enterococcus faecium sagA mutants have cell envelope defects influencing antibiotic resistance and bacteriophage susceptibility
Source: J Bacteriol. 2025 Oct 9;207(11):e00302-25. doi: 10.1128/jb.00302-25 (PMC12632251; doi:10.1128/jb.00302-25)
Supplement: Supplemental tables and figure — Tables S1 and S2, and Figure S1. [file jb.00302-25-s0001.pdf]

Table S1. *sagA* complementation restores ceftriaxone resistance for *E. faecium* 81R6

**Strain**	**MIC (µg/ml)**[a]
E. faecium Com12 (vector[b])	32
E. faecium 81R6 (vector[b])	2
E. faecium 81R6 (p*sagA*[c])	32

[a]Median MICs determined from at least three independent replicates; [b]Vector indicates cells were transformed with the plasmid pAM401; [c]*sagA* complementation plasmid

Table S2. Bacterial strains, plasmids, and phages

Strains	Description	Reference
Enterococcus faecium		
Com12	Human fecal isolate; USA, 2006	(1)
81R6	9181 resistant mutant of Com12; *sagA* insertion; Phe insertion between Tyr 451 and Leu 452	(2)
81R8	9181 resistant mutant of Com12; *sagA* SNP; Gly 435 Val	(2)
81R6-*sagA*	81R6 (*sagA* SNP) strain carrying pAM401-*sagA* complementation vector	(2)
81R6-E	81R6 (*sagA* SNP) strain carrying pAM401 empty vector	(2)
81R8-*sagA*	81R8 (*sagA* SNP) strain carrying pAM401-*sagA* complementation vector	(2)
81R8-E	81R8 (*sagA* SNP) strain carrying pAM401 empty vector	(2)
Escherichia coli		
TG1	*[F' traD36 proAB lacIqZ ΔM15] supE thi-1 Δ(lac-proAB) Δ(mcrBhsdSM)5(rK$^-$ mK$^-$)*	Lucigen
BL21-CodonPlus (DE3)-RIL	*E. coli B F– ompT hsdS(rB$^-$ mB$^-$) dcm$^+$ Tet^r gal l(DE3) endA Hte [argU proL Camr] [argU ileY leuW Step/Spec^r*	Agilent
**Phages**		
9181	Sewage isolate, Siphophage	(2)
**Plasmids**		
pAM401	*E. coli*-*E. faecalis* shuttle vector; pIP501 origin; Cm^R, Tc^R	(3)
pAM401-SagA	pAM401 plasmid expressing *E. faecium* Com15 *sagA* promoter fused to *sagA* ORF with His-6 tag; Cm^R, Tc^R	(4)
pET-21a(+)	N-terminal T7 Tag sequence plus a C-terminal His Tag sequence, Amp^R	Novagen
pET-21a(+)-SagA	Efm_Com15_SagA_∆SS-His6, Amp^R Cm^R	(5)

pET-21a(+)-81R6_SagA-81R6	Efm_Com15_SagA_ΔSS- L insertion between_Y451_L452-His6, Amp^R Cm^R	This study
pET-21a(+)-SagA-81R8	Efm_Com15_SagA_ΔSS-G435V-His6, Amp^R Cm^R	This study
**Primers**	**Primer Name**	**Sequence (5' to 3')**
81R6	SagA-NlpC/p60_L insertion between_Y451_L452_ F 60	TTGTTGCAAGTAACTGGTCGTG
81R6	SagA-NlpC/p60_L insertion between_Y451_L452_R 60	GTAAACATAGCGTGTGAATC
81R8	SagA-NlpC/p60 G435V_F 60	TGTTTGGGGCGTCAAAGATCCAAG
81R8	SagA-NlpC/p60 G435V_R 60	TAAGGAGTACCAATATATTTGTAAG

1. Palmer KL, Godfrey P, Griggs A, Kos VN, Zucker J, Desjardins C, Cerqueira G, Gevers D, Walker S, Wortman J, Feldgarden M, Haas B, Birren B, Gilmore MS. 2012. Comparative genomics of enterococci: variation in *Enterococcus faecalis*, clade structure in *E. faecium*, and defining characteristics of *E. gallinarum* and *E. casseliflavus*. mBio 3:e00318-11. 10.1128/mBio.00318-11

2. Canfield GS, Chatterjee A, Espinosa J, Mangalea MR, Sheriff EK, Keidan M, McBride SW, McCollister BD, Hang HC, Duerkop BA. 2021. Lytic bacteriophages facilitate antibiotic sensitization of *Enterococcus faecium*. Antimicrob Agents Chemother 65:e00143-21. 10.1128/AAC.00143-21

3. Wirth R, An FY, Clewell DB. 1986. Highly efficient protoplast transformation system for *Streptococcus faecalis* and a new *Escherichia coli-S. faecalis* shuttle vector. J Bacteriol 165:831-6. 10.1128/jb.165.3.831-836.1986

4. Rangan KJ, Pedicord VA, Wang YC, Kim B, Lu Y, Shaham S, Mucida D, Hang HC. 2016. A secreted bacterial peptidoglycan hydrolase enhances tolerance to enteric pathogens. Science 353:1434-1437. 10.1126/science.aaf3552

5. Kim B, Wang YC, Hespen CW, Espinosa J, Salje J, Rangan KJ, Oren DA, Kang JY, Pedicord VA, Hang HC. 2019. *Enterococcus faecium* secreted antigen A generates muropeptides to enhance host immunity and limit bacterial pathogenesis. Elife 8:e45343. 10.7554/eLife.45343

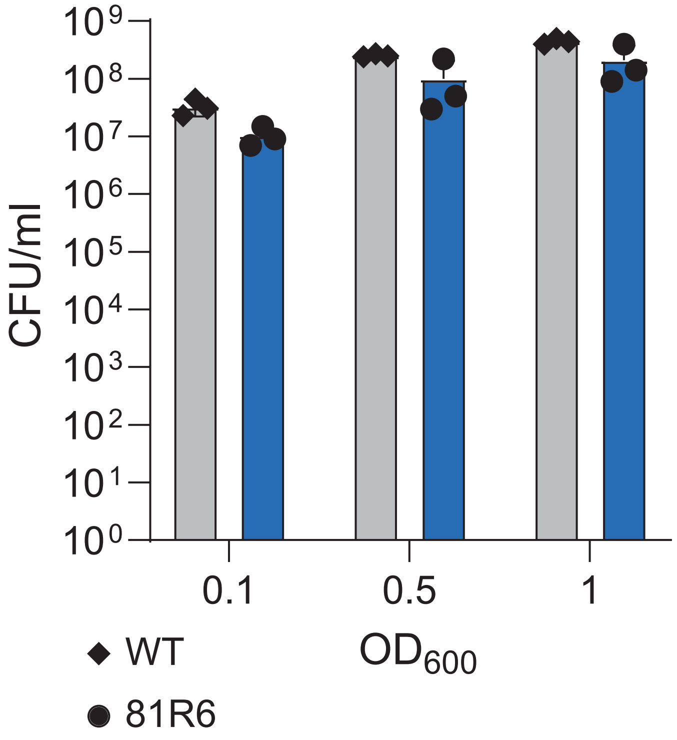

Figure S1. Colony forming units of *E. faecium* strains. *E. faecium* Com12 (WT) and 81R6 were grown in BHI broth. Colony forming units of each strain were determined at OD_{600} of 0.1, 0.5, and 1.0 by plating on BHI agar and incubating at 37°C overnight. Data show three biological replicates per OD_{600} reading.
